# Inhibition of Polo-like kinase 4 induces mitotic defects and DNA damage in diffuse large B-cell lymphoma

**DOI:** 10.1038/s41419-021-03919-x

**Published:** 2021-06-23

**Authors:** Yi Zhao, Juan Yang, Jiarui Liu, Yiqing Cai, Yang Han, Shunfeng Hu, Shuai Ren, Xiangxiang Zhou, Xin Wang

**Affiliations:** 1 Department of Hematology, Shandong Provincial Hospital, Cheeloo College of Medicine, Shandong University, Jinan, Shandong 250021 China; 2 Department of Hematology, Shandong Provincial Hospital Affiliated to Shandong First Medical University, Jinan, Shandong 250021 China; 3 School of Medicine, Shandong University, Jinan, Shandong 250012 China; 4 Shandong Provincial Engineering Research Center of Lymphoma, Jinan, Shandong 250021 China; 5 Branch of National Clinical Research Center for Hematologic Diseases, Jinan, Shandong 250021 China; 6 National Clinical Research Center for Hematologic Diseases, the First Affiliated Hospital of Soochow University, Suzhou, 251006 China

**Keywords:** B-cell lymphoma, Cytokinesis, DNA damage response

## Abstract

Polo-like kinase 4 (PLK4), a key regulator of centriole biogenesis, has recently been shown to play key roles in tumorigenesis. Blocking PLK4 expression by interference or targeted drugs exhibits attractive potential in improving the efficacy of chemotherapy. Nevertheless, the role of PLK4 in diffuse large B-cell lymphoma (DLBCL) is still undefined. In this study, we discover that PLK4 is a potential target for the treatment of DLBCL, and demonstrate the efficacy of a PLK4 inhibitor when used in combination with doxorubicin. Pharmaceutical inhibition of PLK4 with CFI-400945 inhibited DLBCL cell proliferation and induced apoptotic cell death. The anti-tumor effects were accompanied by mitotic defects, including polyploidy and cytokinesis failure. Activation of p53 and Hippo/YAP tumor suppressor signaling pathway was identified as the potential mechanisms driving CFI-400945 activity. Moreover, CFI-400945 treatment resulted in activation of DNA damage response. Combining CFI-400945 with doxorubicin markedly delayed tumor progression in DLBCL xenografts. Finally, PLK4 was increased in primary DLBCL tissues and cell lines. High levels of PLK4 expression were associated with poor survival in the patients receiving CHOP-based treatment, implicating PLK4 as a predictive biomarker of DLBCL chemosensitivity. These results provide the therapeutic potential of CFI-400945 both as monotherapy or in combination with doxorubicin for the treatment of DLBCL.

## Introduction

Diffuse large B-cell lymphoma (DLBCL) is the most common type of lymphoid neoplasm and constitutes 25–35% of non-Hodgkin lymphoma [1]. DLBCL is a phenotypically and genetically heterogeneous disease which has a variable response to therapy [2, 3]. Although addition of rituximab to the standard cyclophosphamide, doxorubicin, vincristine, and prednisone (CHOP) regimens has significantly improved the survival of DLBCL patients, ~30–40% of patients with relapsed or refractory DLBCL have few effective treatment options. The clinical needs for identifying new molecular biomarkers predicting chemosensitivity and more effective targeted cancer therapeutics remain unmet.

One of the most distinguishing hallmarks of human cancer is cell cycle aberrations that lead to uncontrolled cell proliferation and division. Therefore, therapies targeting mitosis have been widely used in cancer for decades. Microtubules-targeting agents, which disrupt microtubule dynamics and block mitotic progression, are the most important antimitotic drugs used in the clinical treatment of both solid tumors and hematological malignancies [4, 5]. However, the clinical utility of microtubules-targeting agents is limited due to severe adverse effects and drug resistance [6]. Recently, inhibitors of mitotic kinase have emerged as efficacious alternative treatment options for patients with solid tumors. Several mitotic kinases including cyclin-dependent kinases, aurora kinases, spindle assembly checkpoint kinases, and polo-like kinases (PLKs) were identified as strong candidates for therapeutic targets [7, 8].

Polo-like kinase 4 (PLK4) represents a unique member of the PLK family and functions as a key regulator of centriole duplication [9–11]. PLK4 is expressed as a low-abundance enzyme in proliferating tissues, and has pleiotropic functions in mitotic progression, including mitotic entry and exit, spindle assembly, and cytokinesis [12–14]. In addition to its mitotic functions, PLK4 has also been involved in multiple biological processes, including cell motility, DNA damage repair, and placental development [15–17]. Growing evidence indicates that PLK4 is overexpressed in many subtypes of cancer and high expression of PLK4 confers poor prognosis [18–21]. Depletion of PLK4 arrests centriole duplication, causes mitotic defects and induces cell death [22]. CFI-400945 is a first-in-class, highly selective inhibitor of PLK4. Previous studies have displayed encouraging preclinical anti-tumor activity of CFI-400945 in solid tumors [21–24]. While results from a phase I, dose-escalation trial demonstrated low response rates of CFI-400945 monotherapy in advanced solid tumors (NCT01954316), further development of this inhibitor is worth anticipating due to good tolerability and absence of toxicities [25]. Moreover, these results have shifted the researcher’s focus toward the identification of the disease-specific cohorts, the evaluation of biomarkers of sensitivity, and the exploration of combination strategies [25].

Hematological malignancies display high proliferative rates and therefore most novel anti-mitotics have shown better efficacy when compared to solid tumors [26–28]. However, the efficacy of PLK4 inhibition with CFI-400945 in hematological malignancies is still largely unknown. In the present study, we reported the preclinical efficacy of CFI-400945 in DLBCL cell lines and xenograft models. CFI-400945 improved chemosensitivity of DLBCL cells by inducing mitotic defects and DNA damage, leading to vulnerability to genotoxic drugs in tumor cells and forcing their death. In addition, we demonstrated that PLK4 was overexpressed in DLBCL, and low levels of PLK4 predicted better survival of patients with DLBCL receiving CHOP-based therapy. These findings provide a therapeutic option of targeting PLK4 with CFI-400945 in DLBCL, and suggest that CFI-400945 in combination with doxorubicin may improve the therapeutic outcome of patients with DLBCL.

## Materials and methods

### Cell culture and reagents

Human DLBCL cell lines LY1, LY3, LY8, U2932, and VAL were cultured in IMDM (Gibco, MD, USA), supplemented with 10% FBS (Gibco) and 1% penicillin/streptomycin mixture. All cells were incubated at 37 °C with 5% CO_2_ in a humidified atmosphere. All human cell lines were authenticated by short tandem repeat analysis and tested for mycoplasma contamination. CFI-400945 (#S7552, Selleck, Shanghai, China), doxorubicin (#S1208, Selleck), nutlin-3 (#S1061, Selleck), and barasertib (#S1147, Selleck) were soluble in dimethyl sulfoxide (DMSO) to the storage concentration at 50 mM.

### Cell viability and cell apoptosis assays

Logarithmically-growing cells were seeded into a 96-well plate at a density of 5000 cells/well for cell viability assay. Cells were treated with drugs at different concentrations or the vehicle DMSO 24–96 h later. 10 μL/well Cell Counting Kit-8 (CCK-8; Dojindo, Kumamoto, Japan) was added to the medium and incubated at 37 °C for 4 h, followed by measuring absorbance at 450 nm by SpectraMax M2 Microplate Reader (Molecular Devices, CA, USA). The combination index (CI) values were calculated using CompuSyn (ComboSyn Inc., New York, NY, USA) according to Chou–Talalay method for drug combinations [29]. CI is a parameter indicating the effectiveness of drug combinations. CI < 1 indicates synergism, CI = 1 shows an additive effect, and CI > 1 represents antagonism.

For apoptosis analysis, DLBCL cells with designed treatment were collected and incubated with Annexin V-PE and 7AAD (BD Biosciences, Bedford, MA, USA) for 15 min. Then cells were subjected to the flow cytometry. The rates of apoptotic cells were acquired on Navios flow cytometer (Beckman Coulter, CA, USA).

### Cell cycle assay

Cells were treated with no-serum medium to synchronize cells. Then the cells were treated with drugs at different concentrations for 48 h. The cells were collected and fixed with 70% ethanol overnight at 4 °C, and stained with PI/RNase Staining Buffer (BD Biosciences) for 15 min. The DNA content was monitored by Navios flow cytometer (Beckman Coulter, CA, USA) and the data was analyzed using FlowJo Version 10.1 software (TreeStar, Ashland, OR, USA).

### Western blot analysis

Western blot analysis was carried out as previously described [30]. Primary antibodies used in western blot were listed as below: PLK4 (#NBP1-33042, Novus, USA), LATS1 (#3477, Cell Signaling Technology, USA), phospho-LATS1 (Thr1079) (#8654, Cell Signaling Technology), YAP (#14074, Cell Signaling Technology), phospho-YAP (Ser127) (#13308, Cell Signaling Technology), cleaved PARP (#5625, Cell Signaling Technology), γ-H2AX (Ser139) (#9718, Cell Signaling Technology), phospho-ATM (Ser1981) (#5883, Cell Signaling Technology), phospho-ATR (Ser428) (#2853, Cell Signaling Technology), phospho-Chk1(Ser345) (#2348, Cell Signaling Technology), phospho-Chk2 (Thr68) (#2197, Cell Signaling Technology), anti-Histone H3 (#4499, Cell Signaling Technology). β-actin was used as a loading control. Chemiluminescent signals were detected using the Amersham Imager 600 imaging system (General Electric, USA). ImageJ software (National Institutes of Health, Bethesda, MD, USA) was used to quantify the protein bands normalized to control.

### Nuclear and cytoplasmic fractionation

Nuclear and cytoplasmic fractionation was performed using NE-PER Nuclear and Cytoplasmic Extraction Reagents (Thermo Fisher Scientific, MA, USA) according to the manufacturer’s instructions. The levels of β-actin and Histone H3 were used as loading controls for the nuclear and cytoplasmic fractions, respectively.

### Bioinformatic analysis

The Basso Lymphoma microarray dataset (GSE2350) was used to analyze PLK4 expression in Oncomine (https://www.oncomine.org) [31]. Gene expression profiles of GSE10846 and GSE53786 were obtained from gene expression omnibus. Bioinformatics analysis of survival outcome correlation with PLK4 levels was performed using Genomicscape (http://genomicscape.com). Positive and negative correlated genes were analyzed by DAVID Bioinformatics Resources 6.8 (https://david.ncifcrf.gov) to perform Gene Ontology (GO) analysis. Association between PLK4 expression and hallmark gene sets was analyzed using Gene set enrichment analysis (GSEA) software (Broad Institute, Cambridge, MA, USA).

### Immunofluorescence assay

DLBCL cells treated with designed concentrations of CFI-400945 or DMSO for 24 h were fixed with 4% paraformaldehyde at room temperature for 15 min. Thereafter, cells were permeabilized with 0.4% Triton X-100 for 10 min. The slides were then incubated in blocking solution at room temperature for 1 h, followed by incubation with primary antibodies against YAP and γ-H2AX overnight at 4 °C and then with Alexa Fluor 488-labeled secondary antibody (Abbkine, Beijing, China). Nuclear was stained with DAPI. Microfilaments were stained with phalloidin (Abcam, Cambridge, MA, USA). The immunofluorescence images were acquired with ZEISS 800 confocal microscope (ZEISS, Oberkochen, Germany). The mean fluorescence intensity was measured using Image‐Pro Plus Version 6.0 software (Media Cybernetics, Silver Springs, MD, USA).

### Clinical samples

The lymph node specimens from 65 newly diagnosed DLBCL patients and 20 reactive hyperplasia patients were obtained from Shandong Provincial Hospital. Samples of patients with reactive hyperplasia were referred as control. Histological diagnoses were established according to the WHO classification [32]. Peripheral blood mononuclear cells from healthy donors were isolated by Ficoll-Hypaque density gradient centrifugation. All samples were obtained following informed consent. The study protocol was approved by the Medical Ethical Committee of Shandong Provincial Hospital.

### RNA isolation and quantitative real-time PCR

Total RNA was extracted using Trizol reagent (TaKaRa, Dalian, China) and reverse transcription reaction was performed using reverse transcription reagents (TaKaRa) according to the protocol. Quantitative real-time PCR (qRT-PCR) was performed with SYBR (TaKaRa) on LightCycler480II system (Roche, Basel, Switzerland). The primer sequences were as follows: PLK4 Forward: 5′-GACACCTCAGACTGA AACCGTAC-3′, Reverse: 5′-GTCCTTCTGCAAATCTTGGC-3′. β-actin Forward: 5′-TGACGTGGACATCCGCAAAG-3′, Reverse: 5′-CTGGAAGGTGGACAGCGA GG-3′.

### Immunohistochemistry

Immunohistochemistry (IHC) was performed using a three-step protocol. Antigen retrieval was performed using 0.01 mol/L sodium citrate buffer (pH 6.0) under high pressure. Slides were then incubated in 3% hydrogen peroxide for 15 min, followed by incubation with normal serum to block non-specific binding. The slides were then incubated with primary antibodies overnight at 4 °C. Negative controls were performed with PBS instead of primary antibody. Following 1 h incubation with biotin-labeled secondary antibody for 1 h at 37 °C, slides were incubated with SABC and DAB. The stained slides were counterstained with hematoxylin. IHC staining was evaluated by two independent observers and scored by the proportion of positive stained tumor cells and the intensity of dye color. The observers were blinded to the group allocation during the experiment. The proportion of positive stained cells was graded as 0 (<5%), 1 (5–25%), 2 (25–50%), 3 (50–75%), and 4 (>75%), and the intensity of color was graded as 0 (no staining), 1 (weak), 2 (intermediate), and 3 (strong). The two scores were added and the protein expression levels of PLK4 were defined using the mean score as a cutoff point. Thus, the specimens were assigned to “low” and “high” group. The integrated optical density value of positive staining for PLK4 was evaluated using Image‐Pro Plus Version 6.0 software.

### Lentiviral transfection

Lentivirus vectors encoding PLK4 or an empty lentiviral vector were constructed from Genechem (Shanghai, China). Lentivirus transfection was carried out according to the manufacturer’s instruction. Target RNAi sequences are as follows: shPLK4#1, caGGGAAGTCTTACACTTTAA, shPLK4#2, CAGTATAAGTGGTAGTTTA, shPLK4#3, TTCTATCTTGGAGCTTTAT. The stably transfected cells were selected and maintained 72 h later in the presence of 2 μg/ml puromycin (Sigma-Aldrich, USA).

### Tumor xenograft model

All mice were maintained in specific pathogen-free conditions and all murine studies were performed in accordance with the principles of the Institutional Animal Care and Research Advisory Committee of Shandong Provincial Hospital. Briefly, 5 × 10^6^ LY8 cells, mixed with 100 μl Matrigel (BD Biosciences) were injected subcutaneously into 5-week-old female SCID/Beige mice (Charles River Laboratory Animal Center, Beijing, China). When the tumor volume reached 100–300 mm^3^, the animals were randomly divided into four groups (*n* = 6/group) using a random number generator (EXCEL) and received treatment. Experiments were not blinded. CFI-400945 (7.5 mg/kg) and the vehicle (corn oil) were administrated once daily by oral gavage (p.o.) for 21 days. Doxorubicin (3.3 mg/kg) and the vehicle (PBS) were delivered intravenously once during the treatment period. To monitor tumor growth in living animals by bioluminescent imaging, the mice were anesthetized using isoflurane and injected intraperitoneally with D-luciferin (150 mg/kg, luciferin potassium salt, #122799, Perkinelmer, USA) 15 min prior to imaging with the IVIS imaging system (Perkinelmer). Tumor size was measured by the digital caliper and the volume of tumor was estimated as follows: *V* = length × width^2^/2. All treatments were stopped after 21 days. The mice were sacrificed, and the tumor tissue was removed for IHC analysis as described above.

### Statistical analysis

All data are presented as the mean ± standard deviation (SD) from at least three independent experiments. All statistical analysis was performed using the SPSS version 22.0 (Chicago, IL, USA) and Graphpad Prism 7.0 statistical software (San Diego, CA, USA). Student’s *t* test was utilized for direct comparisons and analysis of variance (ANOVA) was used for multigroup comparisons. Correlation between PLK4 protein expression and clinicopathological characteristics of DLBCL patients was determined utilizing the two-tailed *χ*^2^ test or Fisher’s exact test. *P* < 0.05 was considered to be statistically significant.

## Results

### CFI-400945 triggers growth inhibition and apoptosis in DLBCL cells

**Fig. 1:**
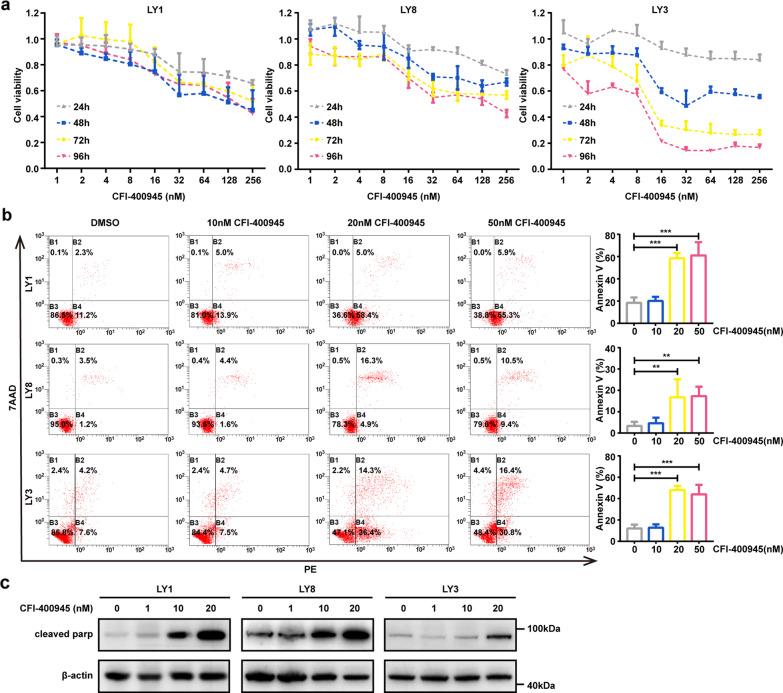
CFI-400945 exhibits potent anti-tumor activities in DLBCL cells. **a** Three representative DLBCL cell lines LY1, LY8, and LY3 were treated with CFI-400945 at various concentrations after 24, 48, 72, or 96 h, respectively. Cell viability was assessed by CCK-8. **b** LY1, LY8, and LY3 cells were treated with increasing doses of CFI-400945 for 48 h. Apoptosis was measured by flow cytometry analysis. **c** The protein levels of cleavage products of PARP were detected in LY1, LY8, and LY3 cells after treatment with CFI-400945 for 48 h. Data are shown as the mean ± SD, *n* = 3. **P < 0.01, ***P < 0.001.

To investigate the effects of CFI-400945 in DLBCL, cells were exposed to increasing concentrations of CFI-400945 and cell viability was detected. CFI-400945 markedly suppressed cell viability in a dose- and time-dependent manner in DLBCL cell lines (Fig. 1a). This effect was higher in *TP53*-unmutated LY3 cell line compared to *TP53*-mutated LY1 and LY8 cell lines [33, 34]. The effects of CFI-400945 on apoptosis in DLBCL cell lines were performed by flow cytometry analysis. CFI-400945 treatment significantly induced apoptosis at concentrations of 20 nM or higher in DLBCL cell lines for 48 h (Fig. 1b). These results were further validated by analyzing the levels of apoptosis-related proteins. As shown in Fig. 1c, CFI-400945 treatment generated higher levels of cleavage products of PARP compared to untreated cells.

### PLK4 inhibition activates p53 and Hippo tumor suppressor signaling pathway by inducing mitotic defects

**Fig. 2:**
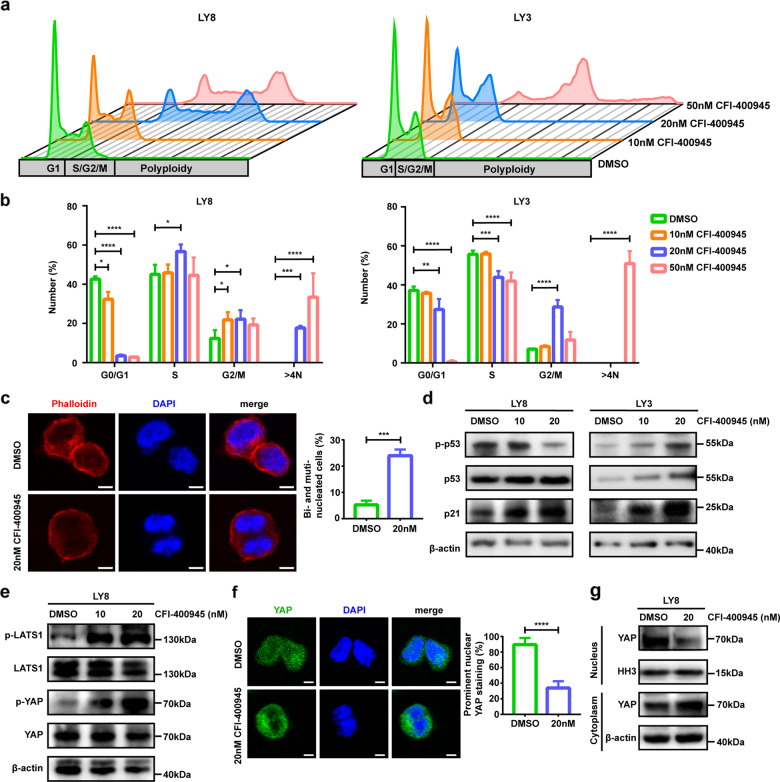
CFI-400945 treatment triggers mitotic defects. **a** Cell cycle profiles of DLBCL cell lines after treatment with 0, 10, 20, and 50 nM CFI-400945 for 48 h. **b** Flow cytometer analysis of DNA content of LY8 and LY3 after treatment with CFI-400945 for 48 h. The proportions of cells in each cell cycle fraction were analyzed. **c** Immunofluorescence staining of Phalloidin and DAPI demonstrated binucleated cells in CFI-400945-treated LY8 cells. Statistics of bi- and multi-nucleated cells were shown on the right. Scale bar: 5 μm. **d** Western blot showed an increase in Serine 15 phosphorylation of p53, accompanied by upregulated expression levels of p53 and p21 upon CFI-400945 treatment. **e** Western blot showed the levels of phospho-LATS1, phospho-YAP, and total-LATS1, total-YAP in CFI-400945-treated LY8 cells. **f** Confocal microscopy showed translocation of YAP from nucleus to cytosol in LY8 cells upon CFI-400945 treatment, with analysis of the percentage of cells with predominantly nuclear YAP shown in the right panel. Scale bar: 5 μm. **g** Nuclear/cytosol fractionation detected by western blot confirmed translocation of YAP upon CFI-400945 treatment. Data are shown as the mean ± SD, *n* = 3. *P < 0.05, **P < 0.01, ***P < 0.001, ****P < 0.0001.

Our finding that CFI-400945 triggers growth inhibition and apoptosis prompted us to explore the underlying mechanism driving CFI-400945 activity in DLBCL. Since PLK4 is involved in mitotic entry and exit, we next examined the effects of CFI-400945 on lymphoma cell cycle progression. Morphologically, lymphoma cells became flatter and enlarged after CFI-400945 treatment for 48 h (Supplementary Fig. S1). For cell cycle analysis, asynchronous growing LY8 and LY3 cell lines were treated with CFI-400945 at increasing concentrations for 48 h. As shown in Fig. 2a, b, the percentage of cells in G2/M phase was increased after CFI-400945 treatment, indicating that a portion of cells were arrested at G2/M. Importantly, an increase in DLBCL cells with DNA content >4 N (polyploidy) was observed at higher drug doses. Confocal microscopy showed that CFI-400945 treatment led to cytokinesis failure and gave rise to binucleated cells (Fig. 2c). These results indicate that PLK4 inhibitor triggers growth inhibition and apoptosis by inducing mitotic abnormalities in DLBCL cells.

Cytokinesis failure has previously been shown to activate tumor suppressor p53 [35]. We treated *TP53*-unmutated LY3 and *TP53*-mutated LY8 cell lines with serial dilution of CFI-400945 for 48 h. Western blot assays revealed that p53 protein is phosphorylated at Ser15 and upregulated in LY3 cells upon CFI-400945 treatment, yet invisibly altered in LY8 cells. An increase in the expression of p21 was observed under the serial increment of CFI-400945 concentrations in both cells (Fig. 2d).

Cytokinesis failure can active the Hippo tumor suppressor signaling pathway [35]. Yes-associated protein (YAP) functions as the key transcriptional regulators that are inactivated by phosphorylation and cytoplasmic localization upon activation of Hippo pathway [36]. We previously established the oncogenic role of YAP in the pathogenesis of DLBCL [37]. Since LY8 cells harbor *TP53* mutation, we proposed YAP as a candidate to drive CFI-400945-induced cytokinesis failure. Here, we found that treatment with CFI-400945 resulted in an increase of LATS1 phosphorylation and consistent YAP phosphorylation after treatment for 48 h in LY8 cells (Fig. 2e). Confocal microscopy showed that CFI-400945 promoted YAP translocation from nucleus to the cytoplasm (Fig. 2f). Western blot assay further confirmed a reduction in the nuclear YAP expression and an increase in the cytosolic YAP expression by nuclear and cytoplasmic fractionation (Fig. 2g). These data suggest that reduced nuclear YAP expression levels and activities contribute to mitotic defects induced by CFI-40045 treatment in DLBCL cells.

### PLK4 inhibition induces DNA damage responses

**Fig. 3:**
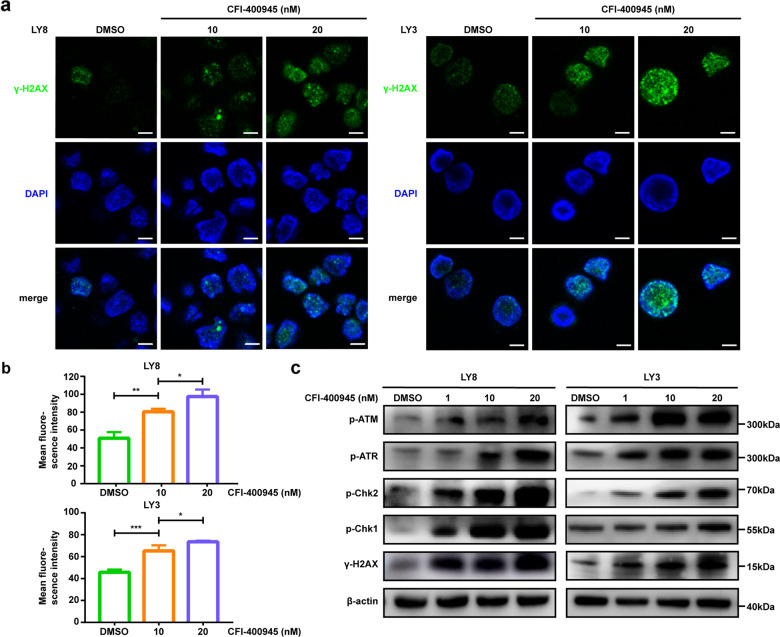
PLK4 inhibition induces DNA damage in DLBCL cell lines. **a** Representative immunofluorescence micrograph showed increased γ-H2AX expression in the DLBCL cell nucleus at 48 h after CFI-400945 treatment. Scale bar: 5 μm. **b** Quantification of γ-H2AX intensity. **c** CFI-400945 activated the DNA damage sensing ATM and ATR pathway. LY8 and LY3 cells were treated with CFI-400945 at the doses indicated for 48 h. Data are shown as the mean ± SD, *n* = 3. *P < 0.05, **P < 0.01, ***P < 0.001.

Recent evidence suggests that polyploid cells generate DNA damage during mitosis [38, 39]. Given the polyploidy and cytokinesis failure induced by CFI-400945, we next investigated whether CFI-400945 treatment-induced DNA damage in DLBCL cells. We exposed cells to different concentrations of CFI-400945 for 48 h and analyzed expression of γ-H2AX, a prerequisite of DNA damage recognition. The confocal immunofluorescent images showed a significant increase of γ-H2AX staining in the DLBCL cell nucleus, indicative of activating DNA damage response (Fig. 3a, b). We next examined the effects of CFI-400945 treatment on other markers of increased DNA damage signaling. Western blot assays showed increased accumulation of phosphorylated ATM (Ser1981) and ATR (Ser428). Chk1 and Chk2 are the kinases downstream of ATR and ATM, and they were shown to be phosphorylated at the Ser317 and Thr68 sites, respectively (Fig. 3c). Thus, these data suggest that DLBCL cells treated with CFI-400945 show increased unrepaired DNA damage, and offer a therapeutic approach that can be combined with genotoxic agents.

### PLK4 inhibitor synergizes effectively with doxorubicin in vitro and in vivo

**Fig. 4:**
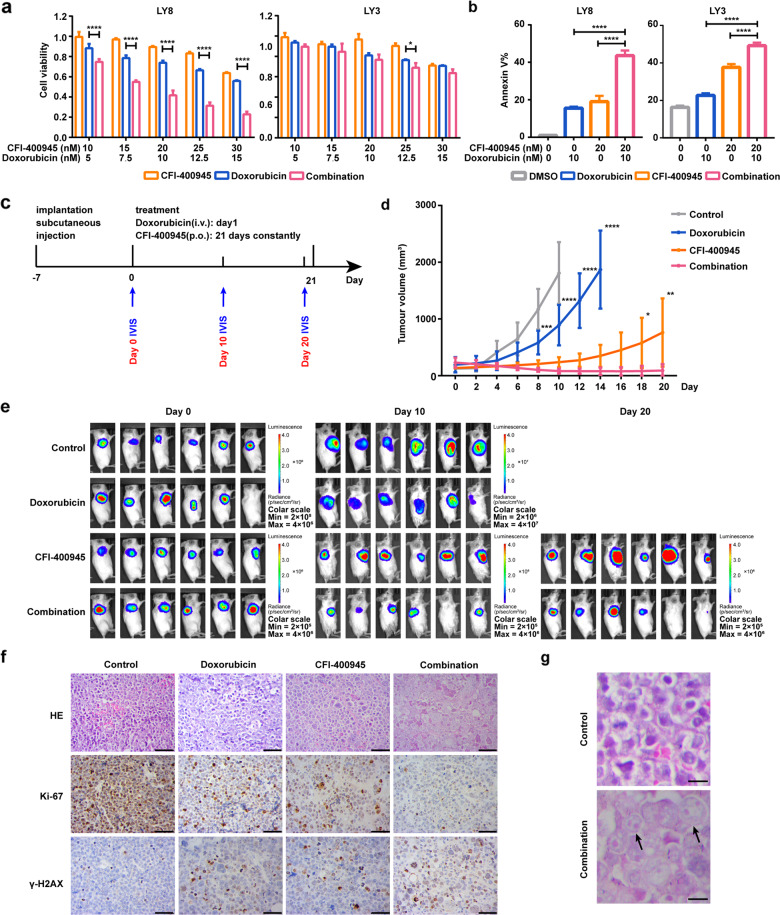
In vitro and in vivo efficacy of CFI-400945 in combination with doxorubicin in DLBCL. **a** Reductions in cell viability induced by CFI-400945, doxorubicin and CFI-400945 and doxorubicin combination in LY8 and LY3 cells after incubation of 48 h. **b** Apoptosis induced by CFI-400945, doxorubicin, and CFI-400945 plus doxorubicin after 48 h in LY8 and LY3 cells. Data are shown as the mean ± SD, *n* = 3. *P < 0.05, ****P < 0.0001 **c** A schema for showing the experimental design of the mice experiment. LY8 cells were injected subcutaneously into SCID/Beige mice. Seven days after injection, mice were treated with CFI-400945 monotherapy, doxorubicin monotherapy or the combination treatment. Doxorubicin was administered on day 1, intravenously, while CFI-400945 was dosed orally and daily for 21 days. Bioluminescent signals were taken at the indicated time points. *n* = 6 for each group. **d** Representative image of tumor growth curves. Data are shown as the mean ± SD, *n* = 6. *P < 0.05, **P < 0.01, ***P < 0.001, ****P < 0.0001 **e** Bioluminescence images of mice from different groups. **f** Representative images of immunohistochemical staining for γ-H2AX and Ki-67 in DLBCL xenograft tumors. Scale bar: 50 μm. **g** Analysis of mitotic status by HE staining to illustrate cells with aberrant mitosis (arrow) in DLBCL xenograft tumors after the combination treatment. Scale bar*:* 10 μm.

We next explored the cytotoxic potential of CFI-400945 in combination with doxorubicin, a frontline genotoxic agent in DLBCL. To evaluate the combined effect of CFI-400945 and doxorubicin on cell proliferation and apoptosis, we treated DLBCL cells with both drugs concomitantly at certain concentrations for 48 h. The results indicated that CFI-400945 synergized with doxorubicin only in LY8 cells, which harbored *TP53* mutation and generated mitotic defects upon CFI-400945 treatment (CI < 1, Fig. 4a and Supplementary Fig. S2). We also detected a synergistic growth inhibition in LY8 cells upon treatment with doxorubicin plus another representative mitotic inhibitor, barasertib, a selective inhibitor of Aurora kinase B (CI < 1, Supplementary Fig. S3a-b). In addition, the apoptosis rate was significantly higher in both cell lines treated with combined treatment compared with CFI-400945 or doxorubicin alone for 48 h (Fig. 4b).

To determine whether CFI-400945 could effectively chemosensitize DLBCL cells in vivo, we evaluated the efficacy of the combined treatment of CFI-400945 and doxorubicin using DLBCL xenograft models. SCID/Beige mice were engrafted with LY8 cells, which were engineered for in vivo imaging as previously described [21]. When tumors reached about 100–300 mm^3^, mice were divided in 4 groups of 6 mice each. Thereafter, we treated the mice with vehicle alone, single-agent CFI-400945 on days 1–21 (7.5 mg/kg, p.o.), single-agent doxorubicin on day 1 (3.3 mg/kg, i.v.) or the combination of CFI-400945 plus doxorubicin (see study design in Fig. 4c). Tumors were continuously monitored followed up by caliper measurements and bioluminescent imaging. CFI-400945 plus doxorubicin increased tumor suppression and growth delay when compared with monotherapies (Fig. 4d, e). We further performed IHC staining of tumor xenografts after treatment in each group. Representative images appear in Fig. 4f. Compared to vehicle control and monotherapy groups, the combined treatment reduced tumor cell proliferation, while increased DNA damage response, as assessed by Ki-67 staining and γ-H2AX staining. Strikingly, tumor cells were generally larger, heterogeneous in size, and frequently binucleated (Fig. 4g). These findings suggest that CFI-400945 enhances anti-tumor efficacy of doxorubicin in DLBCL in vivo.

### Expression profiles and clinical significance of PLK4 in DLBCL

**Fig. 5:**
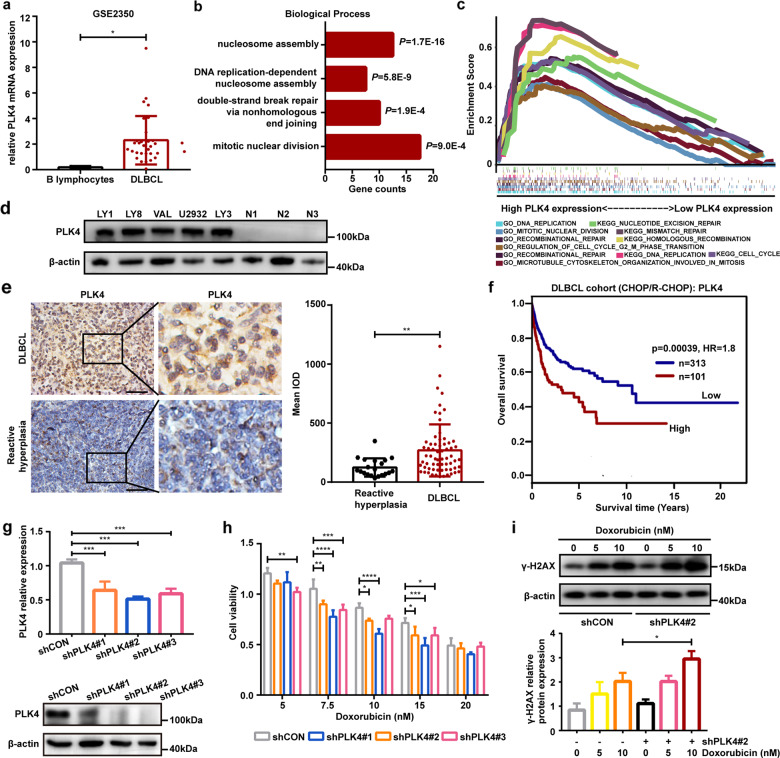
Expression profiles and clinical significance of PLK4 in DLBCL. **a** Gene expression analysis of PLK4 mRNA in primary DLBCL samples (GSE 2350) showed aberrantly increased levels of expression compared with B lymphocytes. **b** GO enrichment analysis of PLK4 co-expression genes in GSE53786. **c** GSEA analysis showed positive correlation between PLK4 expression and DNA replication, cell cycle, and DNA repair. **d** Western blot assays indicated elevated expression of PLK4 in DLBCL cell lines. **e** Representative immunohistochemical images showed high PLK4 expression in DLBCL patients from Shandong Provincial Hospital. Statistics of the integrated optical density value of positive staining were shown on the right. Scale bar: 50 μm. **f** Kaplan–Meier survival curves of DLBCL patients from GSE10846. Data were analyzed through genomicscape (http://genomicscape.com). **g** Lentivirus-mediated RNA interference downregulated PLK4 expression in LY8 cells. Expression of the PLK4 mRNA and protein was assessed by qRT-PCR and western blot analysis. **h** Lentivirus-mediated PLK4 knockdown sensitized DLBCL cells to doxorubicin. **i** DLBCL cells were treated with doxorubicin for 48 h and analyzed for γ-H2AX expression. PLK4-silenced cells showed enhanced phosphorylation of H2AX in a dose-dependent manner. Data are shown as the mean ± SD, *n* = 3. *P < 0.05, **P < 0.01, ***P < 0.001, ****P < 0.0001.

CFI-400945 is a selective inhibitor of PLK4. Thus, we further evaluated expression profiles and clinical significance of PLK4 in DLBCL. Gene expression data were examined using the Oncomine dataset. PLK4 expression was significantly increased in DLBCL compared with the B lymphocytes (*P* < 0.05, Fig. 5a). We next performed functional enrichment analysis of PLK4 in DLBCL using genomic profiles GSE53786. GO analysis demonstrated that PLK4 positive-related genes were enriched in functions including nucleosome assembly, DNA replication and mitotic nuclear division (Fig. 5b). GSEA implicated that PLK4 was functionally enriched in DNA replication, cell cycle, and DNA repair processes (Fig. 5c).

We further analyzed PLK4 expression in DLBCL cell lines. All tested DLBCL cell lines had elevated PLK4 protein expression levels (Fig. 5d). To evaluate PLK4 expression in DLBCL tissues, we performed IHC staining for PLK4 in 65 DLBCL patients. We observed that PLK4 was expressed at higher levels in DLBCL tissues, compared with reactive lymphoid hyperplasia tissues (Fig. 5e). High expression of PLK4 was found in 49 (75%) of 65 DLBCL cases but only 35% (7/20) of the reactive lymphoid hyperplasia tissue samples.

To further investigate the clinical relevance of PLK4 expression in DLBCL, the correlation between PLK4 expression and clinicopathological characteristics were analyzed. High expression of PLK4 was associated with high International Prognostic Index (IPI) score (Table 1). There was no significant difference in PLK4 expression between germinal center B-cell and activated B-cell subtypes. However, multivariate *Logistic* regression showed that PLK4 expression lacked significant correlation with IPI score (Supplementary Table S1). The prognostic significance of PLK4 in DLBCL was also confirmed in public datasets. In a cohort of 414 patients with DLBCL treated with CHOP-based therapy (GSE10846), patients with high PLK4 mRNA levels presented a significantly lower survival rates, compared to those with low PLK4 expression (Fig. 5f).

**Table 1:** Correlation between PLK4 protein expression and clinicopathological characteristics of DLBCL patients.

Variables	No. of patients	PLK4 expression		*P* value
		Positive	Negative	
*Age (years)*				
≤60	36	27	9	0.936
>60	29	22	7	
*Gender*				
Male	30	23	7	0.824
Female	35	26	9	
*Ann Arbor Stage*				
I or II	24	17	7	0.515
III or IV	41	32	9	
*Subtype*				
GCB	20	14	6	0.502
Non-GCB	45	35	10	
*Serum LDH*				
Normal	41	31	10	0.26
Elevated	24	18	6	
*Extranodal involvement*				
Absent	37	26	11	0.271
Present	28	23	5	
*IPI score*				
0–2	30	19	11	0.037*
3–5	35	30	5	

*GCB* germinal center B cell-like, *LDH* lactate dehydrogenase, *IPI* International Prognostic Index.

*P < 0.05.

The above observations prompted us to explore the effects of PLK4 inhibition on chemotherapy in DLBCL cells. Three lentivirus-mediated RNA interference vectors against PLK4 exhibited effective silencing of PLK4 in LY8 cells at the mRNA and protein levels, with shRNA-PLK4#2 demonstrating the highest efficacy (Fig. 5g). PLK4 deficiency led to enhanced doxorubicin-mediated anti-tumor effects (Fig. 5h). Moreover, doxorubicin treatment generated higher levels of γ-H2AX expression in PLK4-silenced cells, as compared to control (Fig. 5i). These results collectively indicate that low levels of PLK4 predict better tumor response to chemotherapy and confirm a potential therapeutic benefit of PLK4 inhibition in DLBCL.

## Discussion

PLK4 plays a key role in centriole biogenesis and centriole duplication. PLK4 overexpression-related centrosome amplification has been implicated as a causative factor for genomic instability and consequent tumorigenesis [40–42]. Elevated expression of PLK4 has been detected in several cancers, and was negatively correlated with chemosensitivity and prognosis of breast cancer and glioblastoma [20, 21]. Our results implicated for the first time that PLK4 was aberrantly upregulated in DLBCL. Low PLK4 levels predicted better response to CHOP-based chemotherapy, suggesting that PLK4 may be a useful biomarker to identify DLBCL patients that respond to conventional chemotherapy. Further validation is warranted to firmly establish the value of PLK4 as a predictive biomarker in clinical practice in DLBCL patients.

Compared to solid tumors, antimitotic drugs show better efficacy in hematological malignancies [43]. Indeed, multiple preclinical and clinical trials have investigated PLK inhibitors in hematological malignancies, especially in acute leukemia, but never in DLBCL [44]. In this study, we focused on the anti-tumor effects of CFI-400945 treatment in DLBCL cells. Results presented here indicated that CFI-400945 treatment significantly triggered potent cytotoxicity through abrogating proliferation and inducing apoptosis in DLBCL cells. LY3 with wild-type *TP53* showed higher sensitivity to CFI-400945 among the DLBCL cell lines studied. Notably, drug combination assays showed that CFI-400945 synergized with p53 stimulation in LY3 cells (Supplementary Fig. S4). However, these findings were not sufficient to support that *TP53* status affected the response to PLK4 antagonism. The role of genetic alterations in the response of DLBCL cells to CFI-400945 deserves further investigations.

It has been indicated that the failure of cytokinesis, leading to the generation of polyploid cells, results in chromosome segregation defects and aneuploidy, and promotes chromosomal instability and tumor initiation [45]. Polyploid chromosomal abnormalities are also associated with worse clinical outcome in DLBCL patients [46]. However, cytokinesis failure has also been suggested to increase chromosomal instability beyond a threshold that induces cancer cell death and was considered as a promising anticancer therapeutic approach. Indeed, several studies have provided promising evidence that promotion of chromosomal instability could be exploited in cancer therapy [47, 48]. In this study, we found that CFI-400945 treatment induced G2/M arrest and polyploidy in DLBCL cells. We also found that CFI-400945 treatment increased cytokinesis failure, which in turn leading to DNA damage and cell death.

Moreover, the major clinical implication of this study is the consideration of combined treatment strategies in DLBCL. Herein, we showed that inhibition of PLK4 by CFI-400945 was directly toxic in DLBCL cells as single agent, but it also sensitized them to genotoxic agents. Combining CFI-400945 and doxorubicin in vivo caused excessive mitotic defects and DNA damage, which in turn suppressed tumor progression. Thus, we propose a mechanism in which CFI-400945 treatment, by causing mitotic defects and DNA damage, promotes synergy with DNA-damaging chemotherapy, exacerbates mitotic abnormalities, and ultimately leads to cell death in response to treatment. In addition, previous studies have shown good tolerability of CFI-400945 in mice at the tested dosage, as shown by loss in body weight < 20%, no significant pathological changes in major organs and tissues, and absence of hematological toxicities other than myelosuppression [21, 22]. The preclinical data presented above indicated CFI-400945 as a promising chemosensitizer in combination with doxorubicin for the treatment of DLBCL. Besides, considering that doxorubicin exerts anticancer efficacy by inhibition of topoisomerase II alpha (TOP2A), our findings suggest that dual inhibition of PLK4 and TOP2A might represent an approach superior to monotherapy. PLK4 is implicated to be co-expressed with TOP2A [14]. It will be of interest to broaden our study to other types of cancers, where TOP2A targeting agents are frequently used.

Notably, recent studies mentioned that PLK4 inhibitor CFI-400945 showed potential off-target effects since the inhibitor also has activity against Aurora kinase B [49]. It has been shown that CFI-400945 is 35-fold more potent in PLK4 inhibition than Aurora kinase B inhibition, as IC50 is 2.8 nM for PLK4 and 98 nM [22]. Consistent with previous studies, the cellular effects of DLBCL cells caused by CFI-400945 treatment peaked at concentrations of 20–50 nM, indicating that this influence was unlikely caused by Aurora kinase B inhibition. Thus, we consider PLK4 as the promising antineoplastic target and the cellular effects by CFI-400945 is likely due to PLK4 inhibition.

In conclusion, we highlight the importance of PLK4 as a therapeutic target in DLBCL and emphasize the need of combining PLK4 inhibitor with genotoxic drugs for cancer treatment. These findings set stage for clinical evaluation of the combination of CFI-400945 and doxorubicin-based chemotherapy in patients with DLBCL.

## Supplementary information

Supplementary table

Supplementary Figure legends

Supplementary Figure S1

Supplementary Figure S2

Supplementary Figure S3

Supplementary Figure S4

## Data Availability

The datasets used and/or analyzed during the current study are available from the corresponding author on reasonable request.
