# Supplementary material for: Inhibition of Polo-like kinase 4 induces mitotic defects and DNA damage in diffuse large B-cell lymphoma
Source: Cell Death Dis. 2021 Jun 23;12(7):640. doi: 10.1038/s41419-021-03919-x (PMC8222327; doi:10.1038/s41419-021-03919-x)
Supplement: Supplementary file 1 — Supplementary table [file 41419_2021_3919_MOESM1_ESM.docx]

**Supplementary Table S1** Multivariate *Logistic* regression of PLK4 protein expression correlated with clinicopathological parameters of DLBCL patients

| Variables | Odds Ratio | 95% CI | | ***P*** value |
| --- | --- | --- | --- | --- |
| **Age (years)** |  |  |  |  |
| ≤ 60 vs > 60 | 0.397 | 0.080 | 1.967 | 0.258 |
| **Gender** | | | | |
| Male vs Female | 0.826 | 0.230 | 2.973 | 0.77 |
| **Ann Arbor Stage** | | | | |
| I or II vs III or IV | 0.687 | 0.143 | 3.306 | 0.64 |
| **Subtype** | | | | |
| GCB vs Non-GCB | 1.503 | 0.417 | 5.415 | 0.534 |
| **Serum LDH** | | | | |
| Normal vs Elevated | 0.956 | 0.234 | 3.906 | 0.95 |
| **Extranodal involvement** | | | | |
| Absent vs Present | 0.524 | 0.071 | 3.851 | 0.526 |
| **IPI score** | | | | |
| 0-2 vs 3-5 | 10.303 | 0.972 | 109.235 | 0.053 |

Note: GCB germinal center B cell-like, LDH lactate dehydrogenase, IPI International Prognostic Index
