# Supplementary material for: Inhibition of Polo-like kinase 4 induces mitotic defects and DNA damage in diffuse large B-cell lymphoma
Source: Cell Death Dis. 2021 Jun 23;12(7):640. doi: 10.1038/s41419-021-03919-x (PMC8222327; doi:10.1038/s41419-021-03919-x)
Supplement: Supplementary file 2 — Supplementary Figure legends [file 41419_2021_3919_MOESM2_ESM.docx]

**Supplementary Figure legends**

**Supplementary Figure S1: Light microscopic images of cell morphology upon CFI-400945 treatment.** LY8 cells were treated with the indicated dose of CFI-400945 for 48 h. Scale bar: 50 μm

**Supplementary Figure S2: CFI-400945 plus doxorubicin synergistically induces growth inhibition in LY8 cells.** The combination index for growth inhibition effects was calculated using Compusyn software.

**Supplementary Figure S3: Barasertib synergizes with doxorubicin in LY8 cells. a** Cell cycle profiles of DLBCL cell lines after a 48-h treatment of with 0 and 10 nM barasertib. **b** Reductions in cell viability induced by barasertib, doxorubicin, and a combination treatment in LY3 and LY8 cells after incubation of 48 h. The combination index calculated by Compusyn is shown on the right. Data are shown as the mean ± SD, *n* = 3. * for *P* < 0.05, ** for *P* < 0.01, *** for *P* < 0.001, **** for *P* < 0.0001

**Supplementary Figure S4: CFI-400945 synergizes with Nutlin-3 in LY3 cells.** Cytotoxic effects induced by CFI-400945, Nutlin-3 and a combination treatment in LY3 and LY8 cells after incubation of 48 h. The combination index calculated by Compusyn is shown on the right. Data are shown as the mean ± SD, *n* = 3. ** for *P* < 0.01, *** for *P* < 0.001, **** for *P* < 0.0001
