# Supplementary figures and images for: Inhibition of Polo-like kinase 4 induces mitotic defects and DNA damage in diffuse large B-cell lymphoma
Source: Cell Death Dis. 2021 Jun 23;12(7):640. doi: 10.1038/s41419-021-03919-x (PMC8222327; doi:10.1038/s41419-021-03919-x)

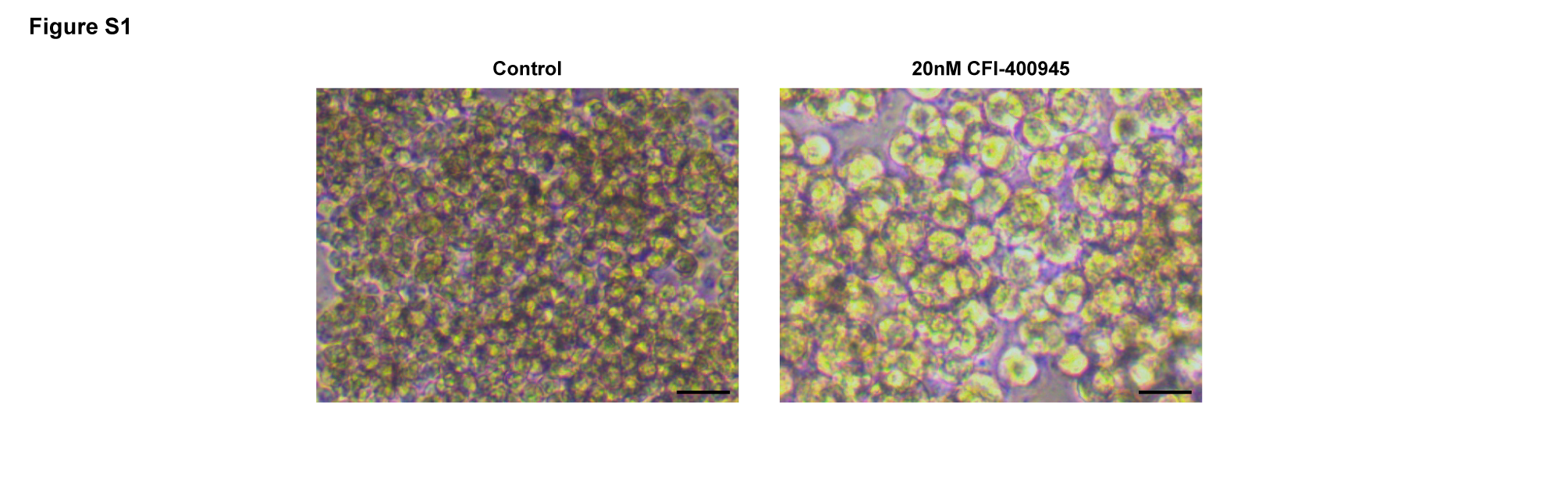

Supplement: Supplementary file 3 — Supplementary Figure S1 [file 41419_2021_3919_MOESM3_ESM.tif]

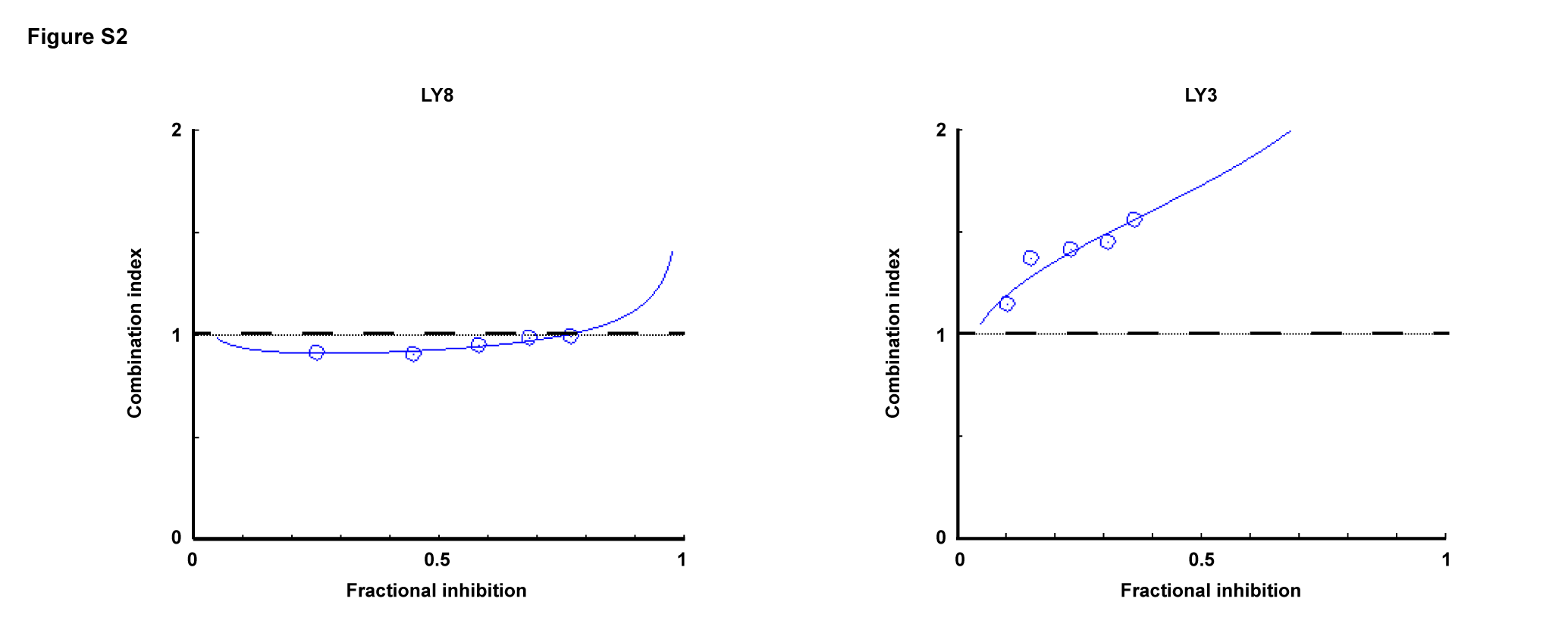

Supplement: Supplementary file 4 — Supplementary Figure S2 [file 41419_2021_3919_MOESM4_ESM.tif]

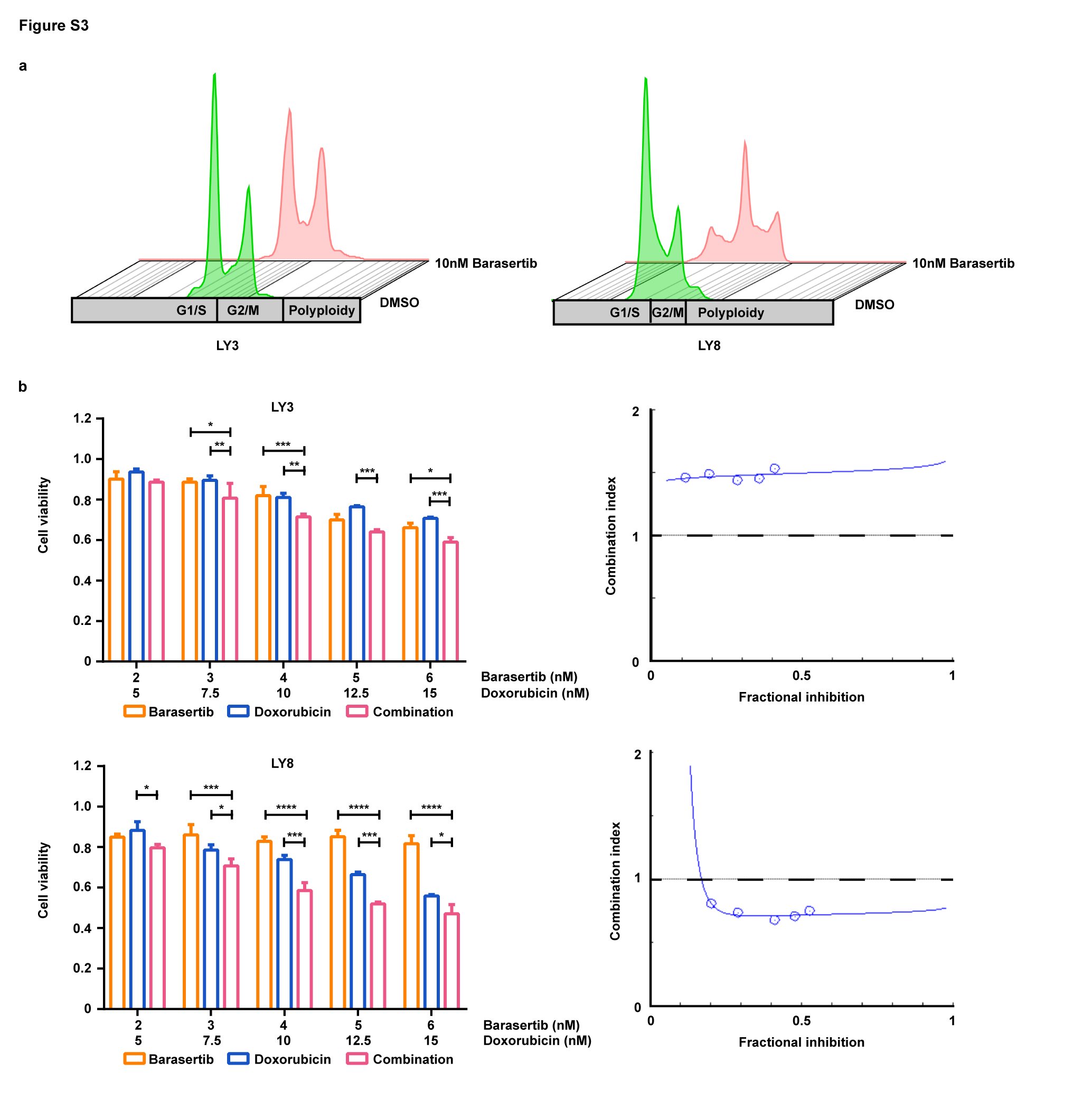

Supplement: Supplementary file 5 — Supplementary Figure S3 [file 41419_2021_3919_MOESM5_ESM.tif]

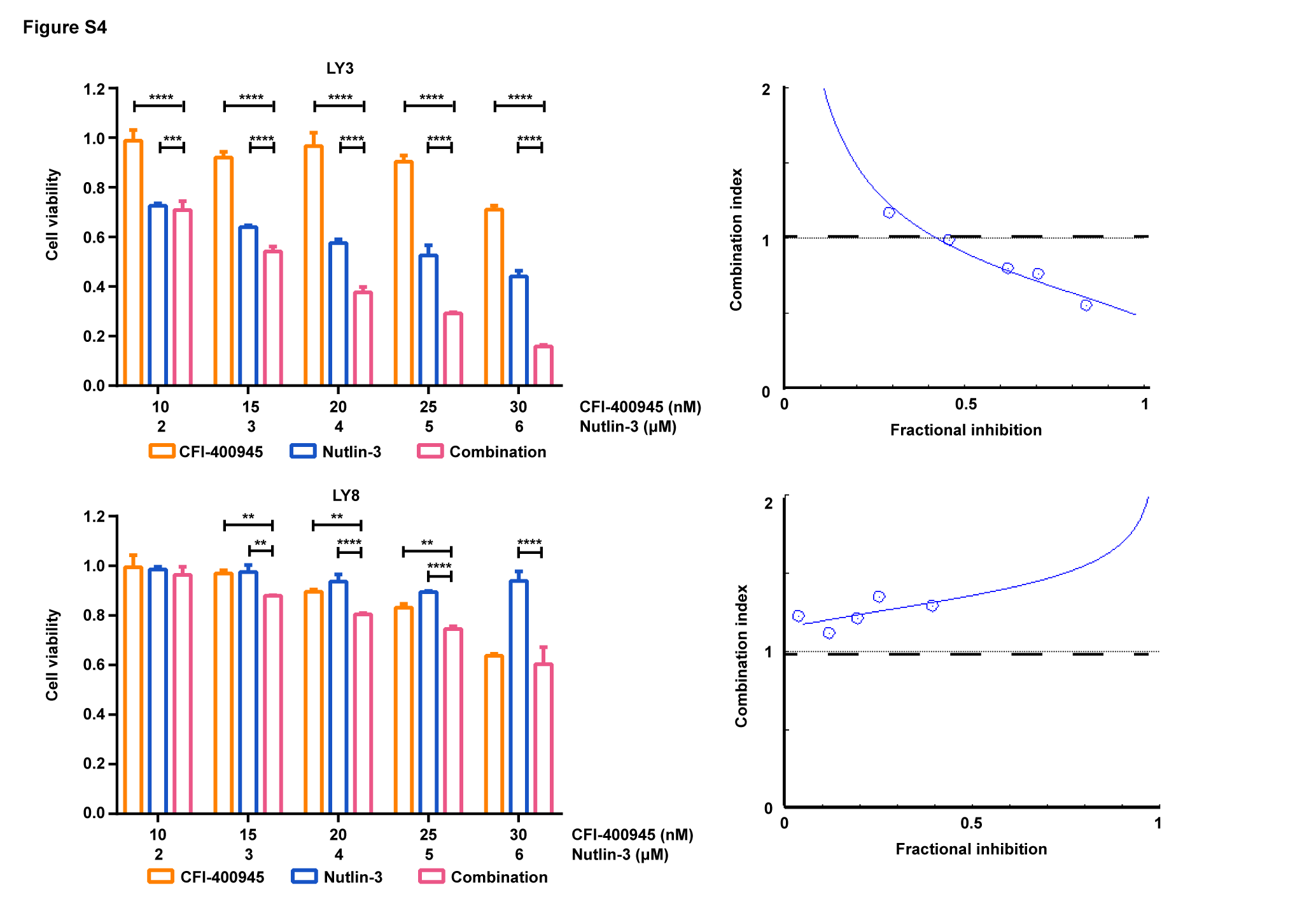

Supplement: Supplementary file 6 — Supplementary Figure S4 [file 41419_2021_3919_MOESM6_ESM.tif]
